# A century of *Hereditas*: from local publication to international journal

**DOI:** 10.1186/s41065-020-00164-8

**Published:** 2020-12-09

**Authors:** Anna Tunlid, Ulf Kristoffersson, Fredrik Åström

**Affiliations:** 1 Department of Arts and Cultural Sciences, Division of History of Ideas and Sciences, LUX, Lund University, Box 192, SE-221 00 Lund, Sweden; 2 Department of Laboratory Medicine, Division of Occupational and Environmental Medicine, Lund University, Scheelevägen 2, SE-223 63 Lund, Sweden; 3 Department of Laboratory Medicine, Division of Clinical Genetics, Lund University, Universitetssjukhuset, SE-221 85 Lund, Sweden; 4 Lund University Library, Lund University, Box 3, SE-221 00 Lund, Sweden

**Keywords:** Hereditas, Mendelian society of Lund, Scientific publishing, Bibliometric analysis, Journal co-citation analysis

## Abstract

**Background:**

The Mendelian Society of Lund launched *Hereditas* in 1920. The purpose of this article is to give an overview of *Hereditas*’s hundred-year existence, focusing on the conditions for a learned society to publish a scientific journal, and the journal’s importance for the publication and dissemination of genetic research. The article focuses on the historical development of the journal and analyses how the content and orientation of research published in *Hereditas* have changed over the years.

**Methods:**

The historical study is based on the collation and interpretation of archival material, mainly held in the Mendelian Society’s archive, which includes the *Hereditas* archive. The bibliometric analyses are based on bibliographic metadata from Web of Science (WoS). Together with descriptive statistics, co-citation analyses were performed by using BibExcel, in combination with the clustering and visualisation tool VOSviewer. Journals with articles citing *Hereditas* articles were identified as a complement to the co-citation analyses.

**Results:**

The history of the journal falls into three main periods: a local period, 1920–1959, when *Hereditas* was primarily intended for Swedish geneticists; a Scandinavian period, 1960–1988, when *Hereditas* was the official journal of the Scandinavian Association of Geneticists; and an international period from 1989 onwards. The original decision that *Hereditas* should cover genetic research with no particular specialisation was retained throughout. Its publications demonstrate the continuing presence of genetic research on plants and animals, albeit with a shifting focus, while human genetics emerged slowly and reached its peak in the period 1960–1988.

**Conclusion:**

In the hundred years of *Hereditas*’s existence, the publishing landscape has changed dramatically, including a far greater number of specialist journals, changes to the academic merit system, new commercial models for publishing, and digitalisation. Over the years, the journal’s survival has therefore been dependent on the strong commitment of its owners and an ability to adapt to changing conditions.

## Introduction

For centuries, scientific publications have served as tools for scientific communication. They have stored and disseminated research results among scientists working in various geographic and intellectual contexts; they have helped to establish and develop scientific networks; and they have served as agents of exchange between learned societies and individual researchers [1]. Scientific journals have played, and are still playing, a crucial role in science communication. Their importance for disciplinary development as well as for individual scientists and their careers can hardly be overlooked.

However, over the long history of scientific publishing, starting in the second half of the seventeenth century with the *Journal de Sçavans* and *Philosophical Transactions*, periodicals and publishing cultures alike have undergone significant changes. This was also true of *Hereditas*, which was founded by the Mendelian Society of Lund in 1920 [2]. Along with a handful of other journals launched in the early twentieth century—the *Zeitschrift für induktive Abstammungs- und Vererbungslehre*, the *Journal of Genetics*, the *Journal of Heredity*, and *Genetics*—it reflected the rapid development of genetics in Sweden as in several other Western countries.^1^ For many years, *Hereditas* was primarily intended for the publication of scientific papers by Swedish geneticists. However, in 1960 it became the official journal of the newly founded Scandinavian Association of Geneticists, and it went on to become a fully international scientific journal in the late 1980s. Today it is an online open access journal issued by BioMed Central (BMC), a publisher owned by Springer Nature. *Hereditas* is thus not only one of the oldest scientific journals of genetics, it has also been part of the changing field of scientific publishing.

This article will give an overview of *Hereditas*’s evolution over its century of existence, focusing on the conditions that pertained for a learned society publishing a scientific journal in the twentieth century, and on the journal’s importance for the publication and distribution of genetic research. It falls into two parts. The first describes *Hereditas*’s historical development as the Mendelian Society of Lund’s chief publication. Since the journal was born out of the local conditions for genetic research, the article will begin with a brief overview of such research in Sweden in the early twentieth century, followed by *Hereditas*’s progression from local publication to international journal. The second part uses bibliometric methods to analyse how the content and orientation of the research published in *Hereditas* have changed over the years. Further, the article is structured around three main periods: 1920–1959, when *Hereditas* was intended as a vehicle for Swedish scientists (and which in turn falls into the interwar period, the Second World War, and the post-war period); 1960–1988, when it was a Scandinavian journal; and 1989–2019, when it became an international journal.

## Material and methods

The empirical material for this article is mainly drawn from the archive of the Mendelian Society of Lund, which includes the *Hereditas* archive. The material for the journal’s early period is comprehensive and publicly available, while there is less material covering the later years, which largely remains in the hands of the Mendelian Society. The historical overview thus centres on *Hereditas*’s early development.

The bibliometric analyses are based on bibliographic metadata from Web of Science (WoS). All the issues of *Hereditas* for the period 1920–2019 were retrieved using the ‘Publication Name’ search field, and the results of the search were downloaded. Descriptive analyses of the data then identified the number of publications per year, the languages of the publications on a yearly basis, and the author affiliation and country for scientists who published in *Hereditas*.

To establish the research orientations of the *Hereditas* articles, co-citation analyses were performed on the references cited in the articles. Co-citation analysis [3, 4] is an established method for mapping research areas, for example the research orientations in a field, and works on the assumption that references cited together in an article share an intellectual commonality. Once aggregated, the number of references cited in a number of publications indicates a specific interest (for example, the current research orientations in a field or, in this case, a journal), which can be visualised using cluster analyses and mapping tools. Co-citations can be analysed at different levels: the publications per se; the cited authors; and the cited journals. When analysing *Hereditas*, journal co-citation analysis was selected [5].

Using BibExcel software for bibliometric analyses [6], the references were extracted from the *Hereditas* publications, specifically as information about the journal in which the citing article was published. This was then analysed to establish the number of times various journals occurred together in the *Hereditas* articles’ list of references. This information was statistically analysed using clustering routines, and was also used for a visualisation technique where co-occurrence frequencies are used as proximity measures in a network analysis. These analyses were performed using a clustering and visualisation tool called VOSviewer [7].

In the resulting ‘map’, the number of citations of a specific journal is represented by the size of the nodes, while the distance between the journals is determined by the co-occurrence frequencies. The more times two journals appear together in lists of references in *Hereditas* articles, the closer they are to each other on the map. The extent to which journals were related to one another was also determined using the clustering routine in VOSviewer, which in the map is represented by the colour of the nodes. For journals represented by the same colour, a statistical relationship has thus been established.

Complementary material for the co-citation analyses was gathered by identifying the most cited publications for each of the periods analysed, and by analysing which journals carried articles citing *Hereditas* publications. This was done using the ‘Create Citation Report’ option in WoS, linked to the publications with references to *Hereditas* articles, which in turn were analysed using the ‘Analyze Results’ option in WoS.

## Genetics in Sweden in the early twentieth century

*Hereditas* was launched at a time when genetics was attracting increasing scientific as well as political attention in Sweden, as in many other Western countries. At plant breeding stations in southern Sweden, a young generation of botanists and breeders started to apply the rediscovered Mendelian laws of heredity in their work, and at Lund University there were lively discussions, mainly among botanists, about Mendelian inheritance, the significance of mutations, and the problems of species formation. They also found inspiration in academic institutions in nearby Copenhagen, particularly the distinguished botanist Wilhelm Johannsen (1857–1927), who was in contact with several of the botanists in Lund. A central figure in these circles was the botanist and geneticist Herman Nilsson-Ehle (1873–1949), who in 1900 started work as a plant breeder at the Svalöf plant breeding station outside Lund. In 1909 Nilsson-Ehle published his doctoral thesis, *Kreuzungsuntersuchungen an Hafer und Weizen* (‘Cross-breeding studies of oats and wheat’), in which he demonstrated that traits with a continuous variation were based on ‘multiple factors’ or genes, and that these genes were inherited according to the Mendelian laws. This was an important development in the theorisation of the Mendelian laws, and achieved wide international recognition.^2^ In 1915 Nilsson-Ehle was appointed professor of botany at Lund University, and in 1917, after a public campaign by colleagues and other proponents of genetics and plant breeding, he was promoted to a personal chair in genetics by the Swedish government, largely in recognition of his success as a plant breeder. In 1925 Nilsson-Ehle was appointed director of the Swedish Seed Association in Svalöv, a position that he was officially allowed to combine with his professorship at Lund University. The university’s Department of Genetics was based in Svalöv, giving Nilsson-Ehle the opportunity to implement his ideas about the mutual dependence of theoretical and applied genetics [8].

Interest in genetics and its practical applications was not limited plant breeding. In the early twentieth century, it was eugenics that increasingly attracted the attention of academics from a variety of disciplines. After several campaigns—among them an exhibition on ‘Swedish racial types’ which in 1919 was shown in several Swedish towns—and protracted political lobbying, the eugenics network succeeded in gaining political support for the establishment of an institute for eugenics. In 1922, the State Institute for Racial Biology, affiliated with Uppsala University, was founded, with the physician and eugenicist Herman Lundborg (1868–1943) as its first director [9, 10]. Lundborg was a close friend of Nilsson-Ehle and both were among the most zealotic proponents of eugenics in Sweden.

When *Hereditas* was launched in 1920, there was a growing Swedish network of geneticists, botanists, and plant breeders who were convinced they represented a new and significant research field. In 1910, they had founded the Mendelian Society of Lund ‘to stimulate interest in modern hereditary research (Mendelism) and related questions’ [2]. The driving force behind the society was the botanist Robert Larsson (1885–1956). He was then working as an assistant at the Department of Botany at Lund University, and was one of the young botanists who in the early twentieth century became deeply interested in heredity, and specifically the new Mendelian theory. Due to health problems (‘weak nerves’ as it was called then), Larsson was not able to complete his academic studies. Instead, he became one of the most important translators, popular science writers, and promoters of the new science of genetics. He made early contact with both Nilsson-Ehle and Herman Lundborg. At the inaugural meeting of the Mendelian Society, Nilsson-Ehle was elected chairman and Larsson secretary. Nilsson-Ehle remained chairman until he retired as director of the Swedish Seed Association in 1939, but Larsson resigned as secretary after only 2 years, although he would soon hold another important post, namely editor of *Hereditas*.

The Mendelian Society became a gathering place for academics, plant breeders, and others with an interest in heredity. It held meetings about current topics in genetics, either taken from scientific journals or from the members’ own investigations. According to the minutes, discussions were often lively.^3^ Sometimes national or international guests were invited to speak on their particular topics. In time, the Mendelian Society became the central platform for the local genetic network in southern Sweden. From 1920 on, the publishing of *Hereditas* was one of the Mendelian Society’s major tasks; indeed, from its inception *Hereditas* was the mouthpiece of a thriving circle of academic researchers and plant breeders associated with the Mendelian Society.

## The early days of *Hereditas*

In early 1919, the Mendelian Society decided to explore the possibility of financing a new scientific journal. It was felt it would be useful for Swedish geneticists to have their own journal where they could quickly publish their results, particularly as it was anticipated that the established scientific journals would be inundated with manuscripts now the First World War had ended.^4^ The subsequent decision to go ahead prompted a flurry of activity to raise the necessary money. A call for financial support was made that emphasised the strong position of Swedish genetic research and the economic and societal benefits to be had, particularly in plant breeding and eugenics.^5^ Companies and individuals with an interest in plant breeding were approached, such as seed companies, mill associations, and breweries.

By the end of 1919, the Mendelian Society considered that sufficient funds had been raised, and they decided the new journal would start publishing in 1920. Robert Larsson was elected editor along with an editorial committee of three: Nilsson-Ehle, the botanist Nils Heribert Nilsson (1883–1955), and the zoologist Gustav Thulin (1889–1945).^6^ At the next meeting, they settled the journal’s name: *Hereditas, Genetiskt Arkiv*. The editorial committee was enlarged by the election of a fourth member, Herman Lundborg—an indication that *Hereditas* was also to include articles concerning human genetics and eugenics.^7^

The editor’s duties and responsibilities were extensive, and included reviewing manuscripts and accepting those consistent with the journal’s remit, overseeing the printing and typographic design, and holding the purse strings. His instructions were to publish manuscripts in the order in which they were received, while rejections should be decided in consultation with the editorial committee. Where manuscripts were deemed too long or too expensive to publish, he had to consult the Society’s board. The board also had to be consulted on all major financial issues. The editor’s had a freeish hand, but his position was spelt out: he was ‘the representative of the Society in everything concerning the journal, and should oversee and protect the society’s interests in this respect’.^8^

**Fig. 1:**
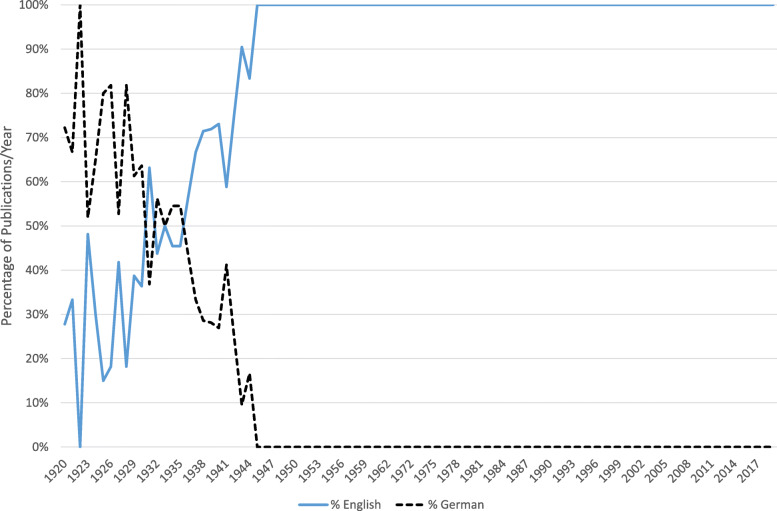
Publication language per year

The first issue of *Hereditas* came out in the spring of 1920. It had eight articles, of which six concerned plant genetics and two human genetics, and all but one were written by members of the Mendelian Society. It had a print run of 1000 copies, while subsequent issues were 750. The journal’s remit was very broad: it was to publish original research about heredity. Articles should be written in English, German, or French, and communicate their findings to the international scientific community. German predominated at first, but was steadily overtaken by English, which was the commonest language by the late 1930s (Fig. 1).

The international aspect of the Mendelian Society’s network followed the same pattern as the languages it published in: German contacts initially dominated, but soon relations with British and American researchers were established. Contacts with American genetics further increased in the interwar years when several of the young geneticists received scholarships from the Rockefeller Foundation and spent their post docs in the US [8]. Although Swedish scientists in most disciplines started to publish in English in the interwar period, genetics stands out with 60% of published pages in Swedish periodicals written in English in 1929–1939, followed by astronomy with 45% of its content in English, and botany with 17%.^9^

*Hereditas* had been founded as a publication for and by Swedish geneticists, partly because of its Swedish funding, partly because of the requirements imposed by government grants (see below).^10^ However, it was not long before the idea of *Hereditas* becoming a Scandinavian journal was mooted. The proposal was made by Johannsen.^11^ After protracted negotiations with representatives from the other Scandinavian countries, the plans were finally abandoned when the Norwegian geneticists announced that they could not contribute financially and therefore found it impossible to participate.^12^ However, although *Hereditas* was not transformed into a Scandinavian journal, articles by Scandinavian researchers were published when space allowed. In exceptional cases, even articles by geneticists outside Scandinavia were accepted. This applied to foreign guest speakers who visited the Mendelian Society and afterwards could have their presentations published in *Hereditas*. Anniversary issues were another exception. When in 1927 the Mendelian Society paid tribute to Johannsen on his 70th birthday with a special issue, it included several articles by prominent foreign geneticists from both Scandinavia and elsewhere.^13^ In this way, *Hereditas* became a tool for developing the Society’s international networks, despite being intended for Swedish geneticists in the first instance.

The journal was funded by a combination of subscriptions, government grants, and income from capital. Before the Second World War, the number of subscriptions rose from an initial 275 to about 400, of which a substantial number were foreign subscribers. Subscriptions were the main source of income for the journal. However, *Hereditas* also received support from the government to covering expenses: after the first few issues, Nilsson-Ehle had applied for government grants on behalf of the Mendelian Society, pointing to the national importance of bringing together the results of Swedish genetic research and making them visible for the international scientific community, as well as the societal and economic benefits of applied research.^14^ The government gave *Hereditas* a publishing grant for its second year, and in subsequent years the Society applied for and received yearly government grants.

The journal’s finances were shaky for many years. It was a constant struggle to keep the publication and distribution costs down while not increasing the price of subscriptions—which might risk losing subscribers—and maintaining the high quality of the journal. For most of the first three decades, *Hereditas* ran at a loss.^15^ It was also affected by the general economic situation, as the Great Depression meant a declining number of subscribers and reduced government funding. The Second World War was if anything even worse in this respect, for due to disruptions to foreign postal services, *Hereditas* lost more than half of its subscribers, just as publishing costs increased and government funding decreased. To rescue the journal, the Mendelian Society had to go cap in hand to private funders.^16^

*Hereditas* existed to disseminate the results of Swedish genetic research to the international scientific community. One way was to get as many overseas subscribers as possible; another, however, was to exchange publications with libraries and other scientific institutions, a standard practice since the eighteenth century. The international publication exchange system has long been a way for libraries to build their collections while facilitating the dissemination of scientific results to the scientific community [11, 12]. Shortly after starting *Hereditas*, the board thus decided to engage in exchanges with other scientific journals if asked.^17^ The reason seems to have been that German scientists and institutions had difficulties paying subscriptions because of the economic crisis and collapse of the exchange rate in the early 1920s. In the interwar years, *Hereditas* was exchanged for 15 scientific journals from various countries, some of which were given to the Department of Genetics at Lund University and some to Lund University Library. The University Library thus did not have to pay to subscribe to these journals, as the Mendelian Society pointed out in its applications for government grants.^18^

Parallel to the publication exchanges, there was also scientific communication on an individual basis. When *Hereditas* was founded, it was decided the authors should not be paid for articles, and instead they should get 100 reprints of their article for free.^19^ Over the years, the Mendelian Society held to its policy, despite the cost of the reprints to the journal. Authors used their reprints in an informal, individual exchange of scientific results with colleagues at home and abroad, as photocopiers or online solutions were far in the future. People’s reprint collections could become very large, testifying to the importance of exchange as a tool for scientific communication. Indeed, reprints were a way for individual researchers to develop their scientific networks.

## From a local to a Scandinavian journal

The immediate post-war period was a time for rebuilding the international scientific relations interrupted by war, but it was also a time for investment in science and technology. It is generally understood that science and technology profoundly affected the course of the war; the development of antibiotics and the Manhattan Project being two of the best-known examples [13]. In Sweden, wartime experiences, both national and international, drove the government to allocate considerable resources to the development of universities and research. As a result, several research councils were established [14].

As part of this, scientific publishing came in for scrutiny. According to the government commission of inquiry that proposed creating the Swedish Natural Science Research Council, all efforts to support science would be inadequate unless measures were taken to get the results published. As the commission emphasised, the publication of science had long faced a difficult situation, as available funds could never cover rising publication costs, especially once scientific production accelerated. The commission therefore suggested scientific publications should receive increased funding, managed by the Swedish Natural Science Research Council.^20^

The funding for scientific journals thus improved, and *Hereditas* continued to obtain publication grants. Its financial situation brightened in other ways too. Several international subscribers had not been able to obtain the journal during the Second World War, and started to buy back issues. In the early 1950s, the sale of past issues was described as ‘abnormal’, which significantly improved the journal’s financial situation.^21^ At the same time, the number of subscribers rose substantially. In 1946, the number was 375 and by 1959 it had doubled, the majority of whom were subscribers outside Scandinavia.^22^ The improved situation meant *Hereditas* started to make a profit, and was able to pay off the debts incurred before and during the Second World War.^23^

There was a major change in 1954 when Robert Larsson resigned as editor, having held the position for 35 years. In that time he had in effect had sole responsibility for the running of the journal. In addition to the long list of responsibilities set out in his instructions when the journal was launched, he was also in charge of distribution, because he considered engaging an external agent to be far too expensive.^24^ For all this work he had been paid a small stipend, decided by the Society’s board.^25^

As editor, Larsson had had great influence over the journal. *Hereditas* had no peer review system in those days. Members of the editorial committee were sometimes consulted, but in most cases Larsson had the opportunity to decide himself what should be published [15]*.* Being a something of a recluse, Larsson ran the journal from his home, rarely leaving other than to visit the printing works. In correspondence with authors and members of the editorial committee, he could be very decided in his views about submissions, both in terms of their content and their style.^26^ However, he could also be very friendly to some authors as the correspondence with Albert Levan (1905–1998) illustrate. Manuscripts Larsson considered good were published quickly, and when planning a new issue of *Hereditas* Larsson sometimes asked if Levan had anything in progress he wanted included.^27^ Being on good terms with Larsson could thus be decisive in getting published. It was a system with very limited anonymity between the author and those who accepted manuscripts for publication.

In 1954, Arne Müntzing (1903–1984), Nilsson-Ehle’s successor as professor of genetics at Lund University and chairman of the Mendelian Society, succeeded Larsson as editor of *Hereditas*.^28^ In the discussions which preceded his appointment, the Board had appealed to Müntzing to take the position, which he did after some consideration.^29^ It was not self-evident that a professor would want to take on the workload of an editor too. A possible reason for doing so was the great importance he and the Mendelian Society ascribed to *Hereditas*. Changes were made to the way the journal was managed. Distribution was entrusted to a bookseller, and a co-editor was appointed to manage the finances.^30^ Decisions about manuscripts were still made by Müntzing, though, assisted by the small editorial committee of four. The original members of the committee were long gone, of course, but as a rule people stayed for very long periods.^31^

Another major change in the post-war era was that the question of transforming *Hereditas* into a Scandinavian journal resurfaced. It followed on the Danish geneticist Mogens Westergaard’s proposal in 1954 to found a Scandinavian society of genetics, following a trend in science and medicine to form such international associations. However, as Westergaard noted, a Scandinavian society would inevitably involve discussions about a joint scientific periodical, and thus raise the question of whether *Hereditas* could be that journal.^32^ The initial reaction by the Mendelian Society’s board was positive, while acknowledging that it raised questions about *Hereditas*’s future which would need careful consideration. The main issue was how to make the journal Scandinavian while retaining the Mendelian Society’s control.^33^ After long negotiations involving representatives from all the countries, the Scandinavian Association of Geneticists was finally founded at a meeting in Copenhagen in 1960 with *Hereditas* as its official journal. According to its statutes, the Mendelian Society was the publisher of *Hereditas* and retained financial responsibility for the journal, and it was the Society which would choose the editor. However, the editorial committee was enlarged. In addition to the four from Sweden, every other Scandinavian country would appoint one member.^34^ As the official journal of the Scandinavian Association of Geneticists, authors from all Scandinavian countries now had the same opportunity to publish in *Hereditas*.

## The Scandinavian period

The 1960s and 1970s were kind to *Hereditas* in terms of publishing. Since the journal was open to submissions from all the Scandinavian countries, the number of issues and published articles increased considerably. Contributions from other Scandinavian countries shot up, although the Swedish articles still dominated. In the 1960s, the number of subscribers increased steadily to stabilise at around 1100, including subscribers in several Western European countries, the US, Canada, India, and Japan.^35^ The journal’s financial situation improved, and no publishing grants were applied for in the decade 1979–1989.

Despite these positive trends, the challenges of publishing became apparent in the 1970s. The growing volume of manuscripts meant far more work for the editor, and more people had to get involved. Apart from Müntzing, there was now a science image editor, Waheeb Heneen, and a secretary (who for many years was Müntzing’s wife), all of them paid a pittance for their services. The system with the editorial committee continued unchanged, and although there were discussions about introducing a system of external referees to complement the editorial board, the Society’s Board considered the pros were outweighed by the cons.^36^ The Board also agreed that *Hereditas* should continue as a Scandinavian journal and not set out to become an international one. By maintaining the Scandinavian focus, it was said, the editor’s assessment of submissions would be significantly easier; however, the Board added, in order to lighten the editor’s load, younger researchers were requested to consult senior researchers about their manuscripts before handing them over to the editor.^37^ It was a standard remark in discussions in the 1970s that the editor often had to do the work which PhD supervisors should have done. That said, even so *Hereditas*’s rejection rate of less than 10% was low, explained by the fact that all the authors were Scandinavians or guest researchers at Scandinavian institutions, and well known to one another.^38^

In 1977, Müntzing resigned as editor and was succeeded by Arne Lundqvist (1920–1998), professor of genetics at Lund University. When Lundqvist took over, it was as part of his university duties as a professor, described as his ‘increased involvement in research supervision’.^39^ At the same time, the editorial board now acquired an editorial secretary, Lars Dävring (1936–2020), who also acted as the image editor. Under an agreement between the Mendelian Society and Lund University, the editorial secretary was employed part-time, indicating that his duties could no longer be considered an unpaid commitment in the service of science.^40^

That Lundquist’s editorship was now counted among his duties as a professor was in line with increasing demands in the 1970s that such commissions of trust ought to be valued as scientifically qualified undertakings.^41^ As Müntzing argued, along with representatives of three other Scandinavian scientific journals, scientific publishing in the region was heading for a crisis. The journals they represented—*Hereditas*, *Oikos*, *Lethaia*, and *Physiologia Plantarum—*were all published by learned societies, and rising costs, increasing internationalisation, and the changing role of editors-in-chief and managing editors had served to make a generally precarious situation worse. The editors’ work was far greater in its scientific, editorial, and technical demands and ate up far more of their time than it ever had before, offered little or no financial incentive, and did nothing to advance their careers: no surprise, then, that it was increasingly difficult to recruit new editors. Faced with this situation, Müntzing and the others suggested that special editorial positions be established where editorships were integrated with research and the supervision of PhD students.^42^ The possibility for Lundquist to hold the editorship of *Hereditas* as part of his chair at Lund University was thus a step in this direction. The arrangement was considered as key reason for *Hereditas*’s healthy finances in the early 1980s, although the Mendelian Society had to pay certain compensation to the Department of Genetics every year.^43^

After the good years, however, *Hereditas*’s fortunes started to slide in the late 1980s. The triggering factor seems to have been falling subscriptions. In the 1980s, *Hereditas* lost some 20% of its subscribers, and the trend accelerated towards the end of the decade.^44^ Such a dramatic fall in revenue would have been disastrous for any journal, and *Hereditas*, according to the Mendelian Society’s statutes, had to be self-sufficient. In addition to the drop in subscribers, the Scandinavian countries were no longer considered to be a sufficient basis for providing the journal with manuscripts.^45^ It was therefore decided that *Hereditas* should be a strictly international journal. At the same time, the editorial structure and management underwent major changes. Karl Fredga, professor of genetics at Uppsala University, was made editor-in-chief in 1989, with Lundqvist, who had retired from Lund University in 1985, continuing as editor alongside Fredga. The editorial secretary was renamed the editorial manager and was made into a full-time position, to include responsibility for distribution. A new editorial board of six was elected, each with specific expertise in different areas of genetics.

Another break with tradition was the appointment of external referees, which meant that for the first time in *Hereditas*’s history it was no longer just the editor or the members of the editorial committee who reviewed submissions for publication. The editorial workload was reduced, but, even more importantly, the system of external refereeing was by this time a well-established practice and considered as a way to strengthen the credibility of scientific publishing [16]. Another change designed to make *Hereditas* more attractive as an international journal was to increase the number of issues per year from four to six, as this would enable a faster publishing rate. The advantage of publishing in *Hereditas*, according to the new policy, would be fast publishing, high production values, and no formal limit on the length of articles.^46^ However, doctoral theses would no longer be given any special advantages. All in all, the changes promised an important renewal of *Hereditas.*

In one respect, Hereditas becoming an international journal with a peer review process was part of the general trend for scientific publications to become part of the academic merit system, not least when assessed using bibliometric indicators, which first became standard in the 1980s, just as international journals with external reviewers evaluating submitted manuscripts became the norm for what constitutes academic publishing [17, 18].

## The international period

The first issue of *Hereditas* in its new format was published in 1989. On the cover there was a call for papers, announcing that *Hereditas* was now ‘a truly international journal’. All submissions would be assessed by two independent reviewers, and the review process would be rapid yet rigorous to insure prompt publication. The ambition was to produce ‘a high-quality journal with original research papers on all aspects of genetics’; the hope was that authors would consider *Hereditas* for their most interesting and important results.^47^

The journal’s renewal entailed large costs, however, and from 1991 *Hereditas* was once again dependent on publication grants. Over the next few years the subscription price was successively raised and the costs of production and distribution were reduced, leading to a better financial situation.^48^

When the plant geneticist and member of the Society, Roland von Bothmer, was commissioned to investigate *Hereditas* in 1994, he concluded that there were good reasons to hope for the future. Its transformation into an international journal had begun to take effect: there had been a shift towards more international articles in recent years. Classical genetics and cytogenetics dominated the content, while there were only a few contributions in molecular biology. The number of subscribers was still falling, but the situation was not precarious. However, the impact factor was quite low (0.616), and *Hereditas* was ranked 59 out of the 70 journals listed under ‘Genetics and Heredity’ by Journal Citation Reports. Despite this, the situation was not considered too alarming compared to equivalent journals such as *Heredity* and *Journal of Heredity.* As von Bothmer argued, *Hereditas* could never achieve the status and impact of more prestigious journals such as *Genes & Development* or *Trends in Genetics*, but should strive to become a good ‘B journal’. Another of his conclusions was that *Hereditas* should continue to be a general genetics journal: any attempt at specialisation would probably mean an initial decrease of subscribers, and the competition with well-established journals in specialised subfields would be stiff. That said, he also concluded there was still a need for major changes to the editorial team, and particularly the organisation of the editorial board.^49^

In the coming years, there were intensive discussions in the Mendelian Society about the future of *Hereditas*. Parallel with that, some changes were already underway. Lundqvist and Fredga retired, replaced as editor-in-chief in 1996 by Ulf Gyllensten, a professor of medical molecular genetics at Uppsala University, indicating that the Society hoped to extend its network of subscribers and potential authors into new disciplinary subfields. The journal was doing well and there were a fair number of subscribers.^50^ Yet it was also becoming increasingly apparent that it was a great commitment for a learned society, in terms of both finance and workload, to be the sole publisher of an international scientific journal. Rising costs of production and distribution, an increasing number of specialist journals to compete with, and reduced opportunities to obtain publishing grants from research councils conspired to make the situation untenable. Several options for *Hereditas*’s future were therefore discussed, from closing down to collaboration with a commercial publisher.

Despite the hurdles, the Mendelian Society recognised the value of continuing to publish *Hereditas* and a number of measures were taken which ultimately led to a new collaboration: in 2002, the Society joined forces with Oikos Editorial Office, an independent publisher at Lund University, which published several biological and ecological journals on behalf of learned societies.^51^ A new editorial manager for *Hereditas*, Petter Oscarsson, was employed to develop the collaboration.^52^ At the same time, the distribution was taken over by the Danish publisher Munksgaard.^53^ Thus, the production and distribution of *Hereditas* were significantly rationalised. In the same year, 2002, Anssi Saura, professor of genetics at Umeå University, succeeded Gyllensten as editor-in-chief.

The collaboration with Oikos Editorial Office went well and the situation improved in several respects; however, the number of subscribers never grew to the point where the journal was financially sustainable. In 2003 it became obvious that something more radical had to be done.^54^ In 2004 the Mendelian Society decided that the publication of *Hereditas*’s paper edition should cease that year. Back issues would be digitised and made available as an online archive.^55^ However, parallel with the decision to end the paper edition, the Board decided to look at whether *Hereditas* could continue as an open access journal, financed by publication fees paid by authors. On further investigation, the Society agreed that *Hereditas* should continue to be published as an online journal ‘in accordance with the Society’s interests and intentions’.^56^ The transition was made when the paper edition ceased. For the Society, it was a great relief that the publisher, Blackwell Munksgaard (later Wiley), showed continued interest in publishing *Hereditas*, thereby avoiding the total closure of the journal.

The shift to online publishing began successfully enough. However, the journal now depended on fees from published articles, and in 2014, Stefan Baumgartner, professor of developmental biology and editor-in-chief since 2012, announced that *Hereditas* had to close due for financial reasons. Too few submitted papers, and consequently too few published papers, had led to a significant financial loss over a number of years [19]. However, sometimes, unexpected things happen. A couple of months after the closure, a new issue of *Hereditas* was published, announcing that the journal was alive and well and had a new publisher, BioMed Central [20]. The reasons BioMed Central gave for their interest was *Hereditas*’s long history and legacy and its affiliation with the Mendelian Society of Lund, which was considered to have a good reputation and visibility. Moreover, the Society and the editors were willing and enthusiastic to develop the journal. In the publisher’s view, taking over *Hereditas* was an opportunity to further strengthen and expand its stable of open access journals.^57^ In all, the stated reasons testify to the mutual interest of the Society and BioMed Central in publishing *Hereditas*, but also to the value of a learned society’s journal to a commercial publisher.

**Fig. 2:**
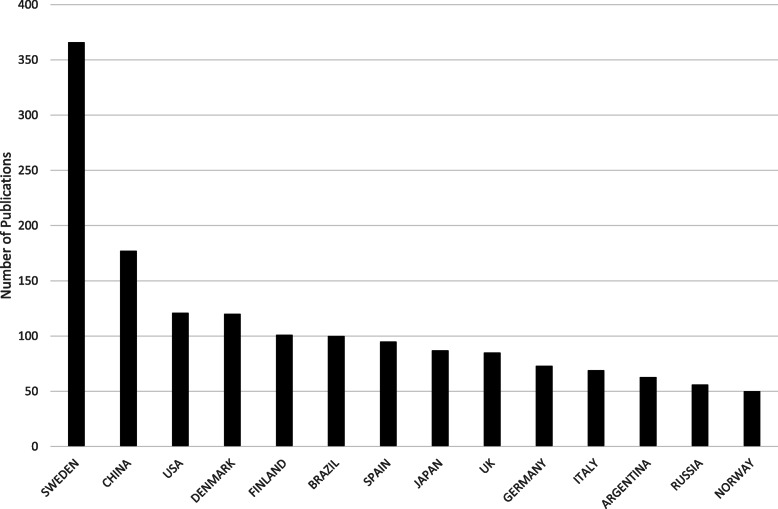
Distribution of publications per author affiliation country, 1989–2019 (≥50 publications)

Several changes have been made since BioMed Central took over, among them the appointment of a second editor-in-chief, Professor Yongyong Shi of Shanghai Jiao Tong University, to share responsibilities with Baumgartner. *Hereditas* now offers a wider selection of articles than previously, and hopes to expand the journal’s activities in Asia and Australia [20]. The new policy has been successful: the number of articles submitted from China forms an increasing part of the journal’s publications (Fig. 2) and the citation index shows a positive trend, with a journal impact factor of 2.412 for 2019. *Hereditas* can thus celebrate its centenary as a truly international journal of genetics.

## Bibliometric and content analyses of *Hereditas*

From the start, *Hereditas* existed to publish original genetic research. The content has been general genetics with no specialisation. However, over the years the orientation of its articles has changed, reflecting the trends in genetic research in Sweden in the interwar period and Second World War (1920–1945) and the post-war period (1946–1959); in Scandinavia (1960–1988); and in the international community of geneticists (1989–2019).

### The interwar period and wartime, 1920–1945

**Fig. 3:**
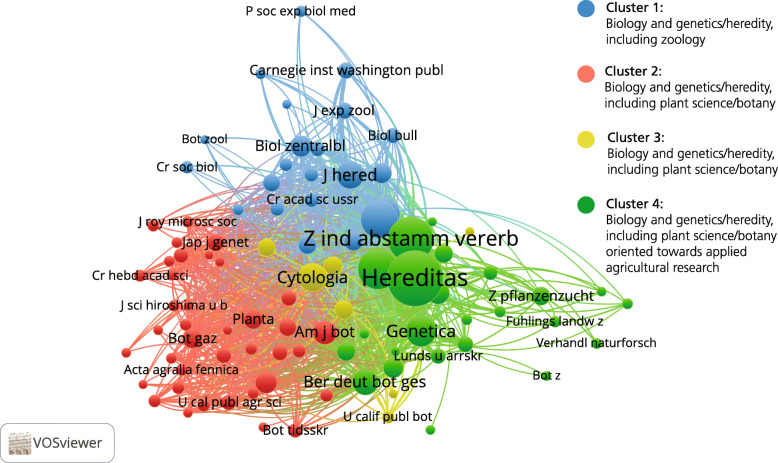
Journal co-citation analysis of references in *Hereditas* articles including 92 journals cited five times or more, 1920–1945

In the analysis of all 8617 references from the 339 articles published in *Hereditas* in 1920–1945, 557 cited journals were identified, of which 92 journals cited at least five times were selected for visualisation and cluster analysis (Fig. 3). The network in itself is relatively tight, with few identifiable structures, suggesting only small variations in the extent to which different journals were cited together. However, the clustering routine draws a clearer distinction in the shape of four different clusters, which also separate the network into two clearly distinguishable sections. In the upper part of the map is a blue cluster with the generalist journals in biology and genetics/heredity as well as the zoology journals. In the lower section are three clusters, one red, one green, and one yellow—albeit less visible. As with the zoology cluster, it includes generalist biology and genetics journals, but there is also a strong group oriented towards botany and plant science: in the case of the ‘red cluster’, with something of an emphasis on general botany, and in the case of the green, with more journals related to applied agricultural research.

Thus we find a distinction between zoology and the plant sciences/botany, where the cluster of cited journals in relation to botany is much larger (in terms of number of journals as well as in times cited in *Hereditas* articles). This suggests *Hereditas* focused on these two aspects of heredity research in the period 1920–1945—primarily botany and to a lesser extent zoological research—whereas its coverage of research on human heredity was almost non-existent. The relative lack of clearly defined structures in the network (meaning the relative proximity between all the clusters) can probably be explained by the strong presence of generalist biology and genetics journals.

A closer look at titles and authors confirms that articles on plant genetics dominated in *Hereditas* in the interwar years and the Second World War. This mirrors the direction of research in Swedish genetics at the time. Several authors were employed at the plant breeding stations in the south of Sweden, but many were also associated with the departments of genetics or botany at Lund University. It should be noted that some of *Hereditas*’s publications were doctoral theses. In the interwar years, twelve of Nilsson-Ehle’s PhD students published their doctoral theses in *Hereditas*, dealing with subjects such as gene ecology, species formation, and cytogenetics. Even during the Second World War, eight theses supervised by Müntzing were published in *Hereditas*, several of them being cytogenetic studies.

**Fig. 4:**
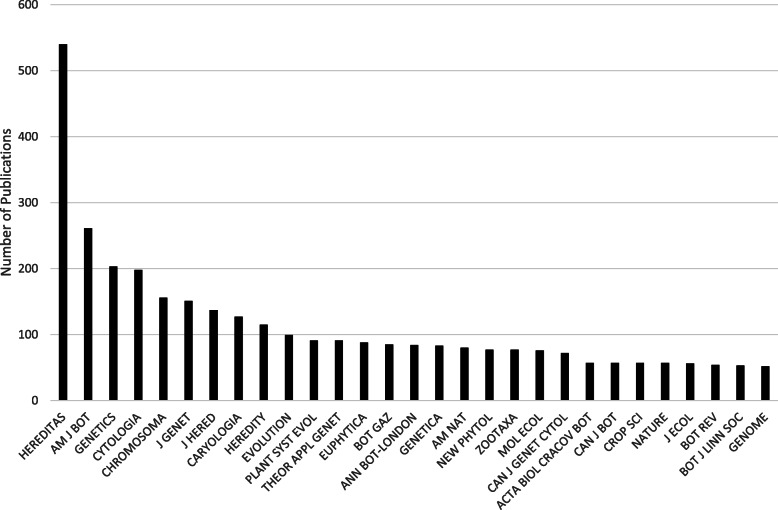
The 29 journals with at least 50 articles citing *Hereditas* publications from 1920 to 1945

In addition to gauging *Hereditas*’s research orientation in 1920–1945 using journal citation analyses, the journals that cited *Hereditas* publications were also analysed (Fig. 4). Of the 29 journals with the largest number of articles that cited *Hereditas* the great majority of the more specialised journals concerned botany, cytology, cytogenetics, and evolution. In the period, the two most cited publications were written by Sten Wahlund and Göte Turesson. Published in the 1920s, they are in fact the two most cited articles in *Hereditas* as a whole, as both have steadily been gathering citations to this day (2020).

The most cited of the two with over 800 citations is Wahlund’s ‘Zusammensetzung von Populationen und Korrelationserscheinungen vom Standpunkt der Vererbungslehre aus betrachtet’ (Population composition and correlation structure considered from the viewpoint of heredity), published by *Hereditas* in 1928 when Wahlund was working at the State Institute for Racial Biology in Uppsala on Herman Lundborg’s large-scale project to measure the ‘racial characters’ of the Sami population in the north of Sweden [21]. As part of this investigation, Wahlund documented the reduction of the overall heterozygosity that occurs when the whole population is divided into sub-populations with different allele frequencies. Such populations are often referred to as being structured. Called the Wahlund effect, it is still being used in population genetic research, as shown by the article being cited some 20–40 times a year in the 2010s.

The second most cited publication was Turesson’s PhD thesis, ‘The genotypic response of the plant species to the habitat’, published by *Hereditas* in 1922, and cited over 500 times. In it Turesson introduced the concept of the ecotype to make a distinction between the hereditary and the ecological aspects of phenotypic variation within species. As with Wahlund’s thesis, Turesson’s is still being cited 10–25 times a year in the 2010s.

### The post-war period, 1946–1959

**Fig. 5:**
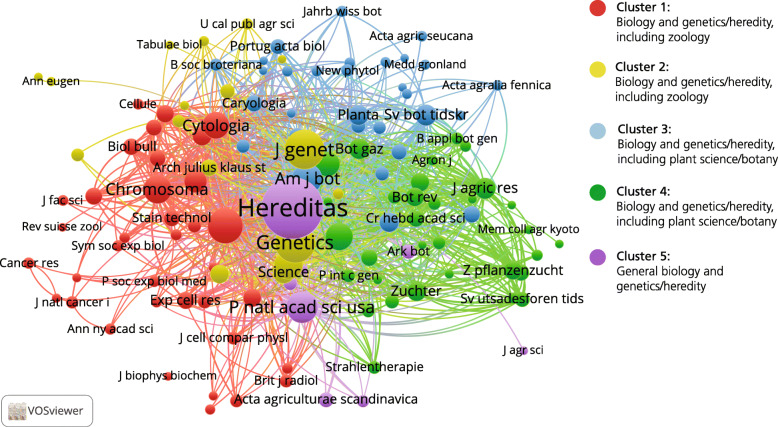
Journal co-citation analysis of references in *Hereditas* articles, including 125 journals cited five times or more, 1946–1959

In the period 1946–1959, there were 332 *Hereditas* publications citing 11,853 references in 787 journals, of which the 125 journals cited five times or more were selected for analysis (Fig. 5). As with the 1920–1945 map, the network per se is relatively tight, with few clear structures identifiable. Again, the cluster analysis helps identify a pattern of red and yellow clusters on the left indicating the presence of zoology journals, and on the right the blue and green clusters of botany journals. In comparison to the 1920–1945 analysis, it is worth noting that the red and yellow zoology clusters include journals related to human genetics and cancer research, such as *Annals of Human Genetics*, *Cancer Research*, and *Radiation Research*. In the analysis of the 1920–1945 publications, human genetics and medical journals were not found among the analysed journals, but here we can see them appearing, albeit without forming any clusters of their own.

**Fig. 6:**
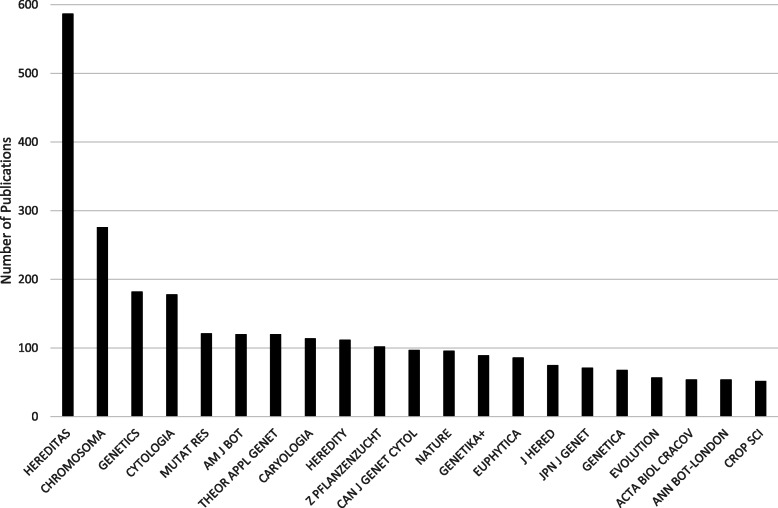
The 21 journals with at least 50 articles citing *Hereditas* publications from 1946 to 1959

Compared to the interwar period, publications concerning cytogenetics increased. Several of those that frequently cited articles in *Hereditas* in this period were cytology and cytogenetics journals such as *Chromosoma*, *Cytologia*, and *Caryologia* (Fig. 6). Moreover, some other topics became increasingly prominent. One of the most notable was mutation research—represented by the journal *Mutation Research*—a fast-growing subfield in genetics in the post-war era, and covering both theory and application. In Sweden, mutation research was carried out at several departments, which published their results in *Hereditas*. At the Forestry Research Institute in Stockholm, Professor Åke Gustafsson led a large interdisciplinary mutation research group, primarily dealing with plants. At the Department of Genetics at Stockholm University, mutation research on *Drosophila* was a major field: introduced by Gert Bonnier, who was appointed professor in genetics in 1936, it was further developed by K. G. Lüning, who succeeded Bonnier as professor in 1958. The appearance of mutation research among the journals that cited *Hereditas* articles should also be seen in relation to the radiation research journals in the red zoology cluster in the map of journals cited in *Hereditas* articles in the period (Fig. 5).

However, the most cited publication in the immediate post-war period—cited some 270 times—concerned a different subfield altogether. In 1956 in their article ‘The chromosome number of man’, Joe Hin Tjio and Albert Levan were the first to show that the human chromosome number was 46, not 48, which until then had been the accepted view. The article was widely recognised and the results, including the cytogenetic method it was based on, played an essential role in the development of medical genetics.

Reflecting on *Hereditas*’s history up to 1959, the importance of genetics for eugenics (race biology) was often emphasised in early descriptions, yet the actual number of articles that could be classified as human genetics, including eugenics, was very limited: only 5% of all articles in 1920–1959 were concerned with studies of human genetics, and the majority of them were empirical studies in population genetics, using descriptive studies of Swedish populations. Only a minority of the studies, mostly published in the early 1920s, were race biological interpretations of data by scientists affiliated with Uppsala University and/or the State Institute for Racial Biology in Uppsala. The clinical case reports were mainly descriptions of families based on observations of dominant hereditary traits.

### The Scandinavian period, 1960–1988

**Fig. 7:**
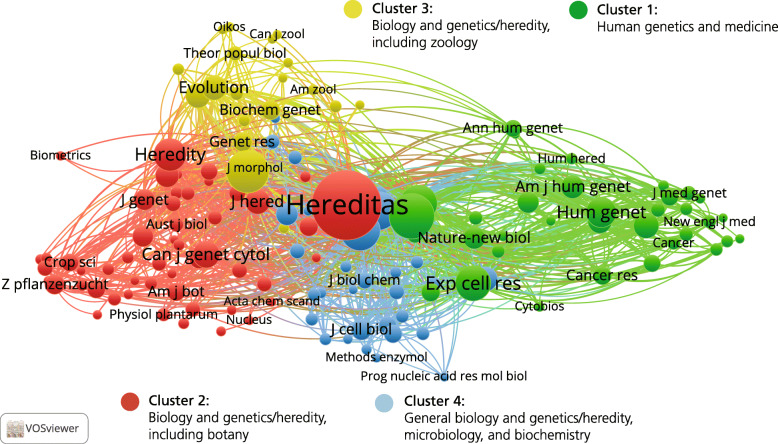
Journal co-citation analysis of references in *Hereditas* articles, including 137 journals cited twenty times or more, 1960–1988

The analysis of *Hereditas* publications in 1960–1988 is based on 2007 publications, citing 40,936 references in 2251 journals. The network and cluster analyses considered the 137 journals cited at least 20 times (Fig. 7). Compared to the previous periods, the network now displays more identifiable structures, even without including the results of the cluster analyses. On the right side of the map are several journals related to human genetics and medical research, which is further emphasised by the cluster analysis. On the left side, there is less distinction between the journals identified as different by the cluster analysis, but at the same time the cluster analysis draws a clear distinction between botany journals in the red cluster in the lower part of the map and the yellow cluster of zoology journals in the upper part. In the middle there is also a blue cluster of generalist journals, primarily related to biology, microbiology, and biochemistry.

One immediate observation is the existence of a separate cluster of human genetics and medical research, with something of an emphasis on cancer research. As for the increasing clarity of the structure of the map, with identifiable structures in terms of the separation between the various research areas, it could be read as indicating the growing significance of specialist journals.

**Fig. 8:**
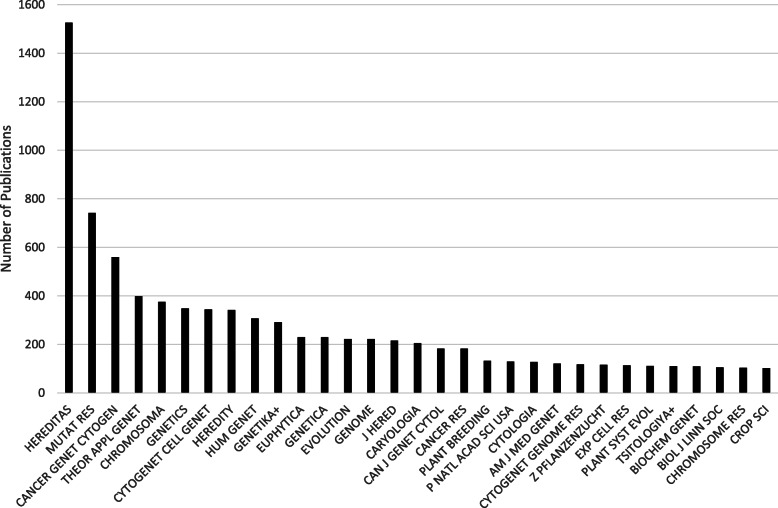
The 31 journals with at least 100 articles citing *Hereditas* publications from 1960 to 1988

This was a period when articles about mutation research were frequently cited by other journals (Fig. 8). A new and rapidly growing research field was cancer genetics, as is reflected in the journal citation analysis map, which shows journals of human genetics and medical genetics—including cancer research—starting to form a cluster of their own. One such journal was *Cancer Genetics and Cytogenetics* (*Cancer Genetics* as of 2011), an American journal founded in 1979 with Avery A. Sandberg as editor-in-chief, which had close connections to Scandinavian research: five of its twenty-eight-strong editorial board came from Sweden or Finland: Albert Levan, his son Göran Levan, Joachim Mark, Felix Mitelman, and Albert de la Chapelle.

This was the golden age for publishing about human genetics more generally. It coincided with the great achievements in cell and molecular biology such as the development of the phytohaemagglutinin method to study chromosomes in human lymphocytes, chromosome banding techniques, and the first attempts in molecular genetics to use Southern blotting. Furthermore, the growing interest in environmental mutagenesis, where many of the leading scientists were based in Scandinavia, led to numerous experimental and clinical studies of the effect of chromosome breaks in human cells. This, combined with the lack of specialist journals and the tradition of publishing in national journals, led to a greater interest in publishing in *Hereditas*. The number of human genetics articles tripled in 1960–1988 compared to 1920–1945.

Yet while the overall trend in this period reflected the rise of human genetics, the most cited publication concerned the monitoring of toxic substances: Geirid Fiskesjö’s ‘The Allium test as a standard in environmental monitoring’, published in 1985 and cited almost 500 times since then. Albert Levan introduced the Allium test in his investigations of the effects of colchicine on chromosomes and mitosis, which had been published in *Hereditas* in 1938. Since then, the test had been frequently used, for instance by Levan and other researchers when studying the cytogenetic effects of various chemicals. In the Fiskesjö article, the test was further developed and applied as a method for environmental monitoring. The article, which was one of several articles in *Hereditas* in this period that dealt with the effect of environmental pollutants on chromosomes, is an example of how an increasing concern about environmental problems has had an influence on *Hereditas*’s content.

### The international period, 1989–2019

**Fig. 9:**
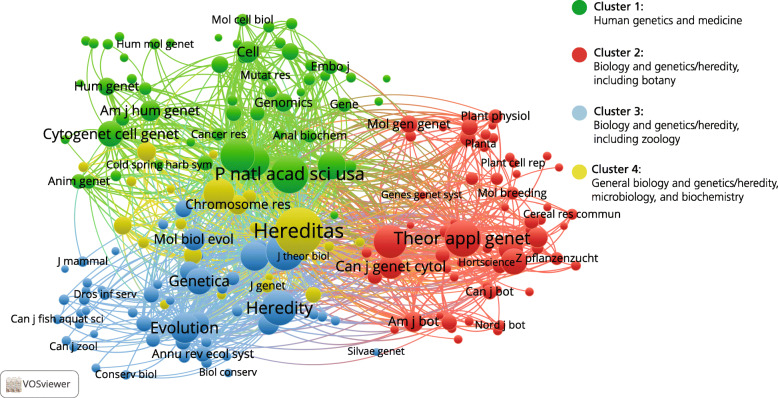
Journal co-citation analysis of references in *Hereditas* articles, including 196 journals cited twenty times or more, 1989–2019

To analyse the 1621 *Hereditas* articles published in 1989–2019—and the 61,620 cited references in 3892 journals—the 196 journals cited 20 times or more were selected for network and cluster analyses (Fig. 9). The network structure with three separate parts to the map has become even clearer than for 1960–1988, and the cluster analysis corresponds to the network analysis. In the upper part of the map, there is a green cluster of human genetics and medical journals; in the lower right part of the map, a red cluster with botany and plant science journals; and in the lower left part, a blue cluster of zoology journals.

**Fig. 10:**
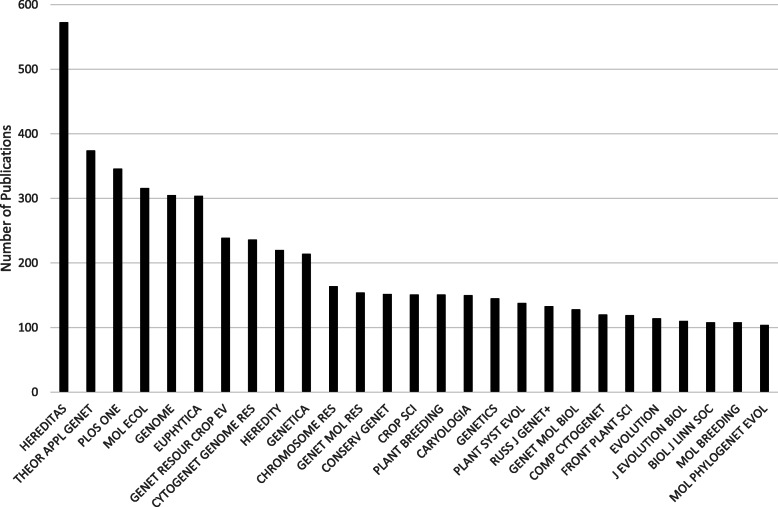
The 27 journals with at least 100 articles citing *Hereditas* publications from 1989 to 2019

The journal citation analysis reveals a wide range of journals, including several new ones (Fig. 10). The number of specialist journals increased, presumably affecting researchers’ choices about where to publish. For example, there was a dip in the number of publications in *Hereditas* related to human genetics, including cancer research, and consequently there will have been few articles in *Hereditas* for the journals specialised in human genetics and cancer to cite. This can be compared to the journal co-citation analysis for the same period (Fig. 9), which also demonstrates the increasing importance of specialist journals. At the same time, we also see how for example *PLOS ONE* joins the journals that frequently cite *Hereditas* articles. *PLOS ONE*, founded in 2006, is an open access journal that is also a so-called mega-journal, publishing thousands—and since 2011, tens of thousands—of articles a year in all fields of science and medicine.

The top-cited publication in *Hereditas* in this period was Moritz’s ‘Conservation units and translocations: Strategies for conserving evolutionary processes’, published in 1999 and cited some 270 times. This article is primarily a survey of the field of conservation genetics, in which Moritz concentrates on the important central question of if and when individuals should be translocated from stable populations to small and therefore threatened populations. The reason for translocations is both to compensate for demographic fluctuations and to reduce the inbreeding effects that occur in small populations. Once again, the top-cited publication of the period relates to a pressing current issue, this time the conservation of biodiversity.

## Conclusions

*Hereditas*’s hundred-year history reflects the enormous changes in the publishing landscape. The number of publications has increased exponentially, digitalisation has fundamentally changed how journals are published, and academic publishing has become increasingly commercialised, just to name a few of the major changes [16]. The editor’s role and the peer review system have also changed beyond recognition in the process [22, 23]. As the publisher of *Hereditas*, the Mendelian Society has had to cope with all these changes and more. It has been challenging at times, but the Society has nevertheless managed to handle it, which testifies to its strong commitment to *Hereditas*.

Although the Mendelian Society has strong local roots in the various institutions in southern Sweden working on genetics, it has always had an international focus. This has been of great importance for *Hereditas*. The point of its existence has always been to disseminate research results to the international scientific community, and, as the journal citation analysis demonstrates, articles published in *Hereditas* have been cited in both generalist and more specialised international journals. However, despite the growing importance of specialist journals, *Hereditas* seems to have preserved its character as a generalist journal with broad scope and aims.

There were evident changes in the network and cluster analyses over time. In terms of the maps, there is an increase of identifiable structures, going from one tight network that included most journals cited in *Hereditas* to a structure of three largely distinct networks with research on plant, animal, and human genetics and heredity. At the same time, the distinction between the different kinds of research has been clearly identified by the cluster analyses. Whereas the increasing importance of specialist journals can safely be assumed, any conclusion that the increase in identifiable structures in the network analysis reflects an increase in specialisation among the authors of *Hereditas* articles would be tenuous. The issue of specialisation should also be considered, bearing in mind the Mendelian Society’s decision that *Hereditas* should remain a generalist genetic research journal and not specialise in one particular aspect of genetic research.

There is a safe conclusion to be drawn about the research presented in *Hereditas*, and that is about the gradual emergence of human genetics from a smaller presence within a larger cluster in immediate post-war period to the formation of its own, clearly defined cluster from 1960 onwards. However, since 1989 there has also been a tendency for human genetic research to be less of a focus.

## Data Availability

The archive of the Mendelian Society of Lund, including the *Hereditas* archive, is held by Lund University Library, Lund, Sweden. Data that support the bibliometric analyses in this study are available from Web of Science/Clarivate, but restrictions apply, and the data which were used under licence for the current study are not publicly available. Data are available from the authors on reasonable request and with Clarivate’s permission.

## Abbreviations

ACS: Arkivcentrum Syd
ADG: Archive of the Department of Genetics
AMA: Arne Müntzing’s Archive
AMS: Archive of the Mendelian Society of Lund
LUB: Lunds Universitetsbibliotek (Lund University Library)
MS: Mendelska sällskapet i Lund (Mendelian Society of Lund)
NFR: Statens Naturvetenskapliga forskningsråd (Swedish Natural Science Research Council)
NOP-N: Nordiska publiceringsnämnden för naturvetenskap (the Scandinavian Publishing Board in Science)
SOU: Statens offentliga utredningar (Swedish Government Official Reports)
WoS: Web of Science

## Footnotes

1 *Zeitschrift für Induktive Abstammung und Vererbungslehre* was founded in 1908 by the German geneticist Erwin Baur; *Journal of Genetics* in 1910 by the British geneticists William Bateson and Reginald Punnett; *Journal of Heredity* in 1914 (in 1910–1913, published as *American Breeder’s Magazine*) by the American Genetic Association; and *Genetics* in 1916 by leading American geneticists, among them G. H. Shull.

2 Independently of Nilsson-Ehle, in 1910 the American geneticist Edward M. East published an article on the inheritance of quantitative traits in which he discussed Nilsson-Ehle’s experiments and results at length.

3 Lunds universitetsbibliotek (Lund University Library), Lund (LUB), Mendelska sällskapet i Lunds arkiv (Archive of the Mendelian Society of Lund) (AMS), vol. 2, Protokoll vid Mendelska sällskapets sammanträden 1910–1940 (Mendelian Society of Lund minutes) (MS Minutes) [transcripts].

4 LUB, AMS, vol. 2, MS Minutes, 16 January 1919.

5 LUB, AMS, vol. 5, S. H. T. (Call for financial support) [1919].

6 LUB, AMS, vol. 2, MS Minutes, 19 December 1919.

7 LUB, AMS, vol. 2, MS Minutes, 23 January 1920.

8 LUB, AMS, vol. 5, Instruktioner för redaktören (Instructions for the editor).

9 Statens offentliga utredningar (SOU) (Swedish Government Official Reports) SOU 1946:77 *Förslag till reformer rörande doktorsavhandlingar och annan publiceringsverksamhet inom naturvetenskaperna: Den marinbiologiska forskningen. Utbildningen av laboratoriebiträden* (Stockholm, 1946), 24.

10 LUB, AMS, vol. 5, Tidskriftens program (The journal’s programme); LUB, AMS, vol. 4, MS Board minutes, 22 April 1920; LUB, AMS, vol. 6, Carl Hallqvist to Øjvind Winge, 31 January 1921.

11 LUB, AMS, vol. 4, MS Board minutes, 22 October 1921.

12 LUB, AMS, vol. 4, MS Board minutes, 5 May 1923; LUB, AMS, vol. 2, MS Minutes, 2 November 1923.

13 Among the authors were Thomas H. Morgan (US), Edward M. East (US), George H. Shull (US), Carl Correns (German), Erich Tschermak von Seysenegg (Austria), Otto L. Mohr (Norway), Harry Federley (Finland), and Øjvind Winge (Denmark) (*Hereditas*, 1927, vol. 9).

14 LUB, AMS, vol. 21, Till Konungen (Application for government grant), January 1921.

15 LUB, AMS, vol. 21, Yearly applications for government grants, 1921–1939.

16 LUB, AMS, vol. 21, Application to Knut and Alice Wallenberg Foundation, 20 November 1944.

17 LUB, AMS, vol. 4, MS Board minutes, 17 November, 23 November 1920; see also LUB, AMS, vol. 5, ‘Da wir von verschiedenen’, 23 November 1920, which was appended to the minutes.

18 One precondition for receiving government grants was that journals had to distribute copies free of charge to numerous government institutions, which many journals felt was a financial drain and reduced the value of any grant (SOU 1946:47, 29–32).

19 LUB, AMS, vol. 4, MS Board minutes, 19 January 1920.

20 SOU 1946:47, 58–60.

21 LUB, AMS, vol. 39, Till Statens naturvetenskapliga forskningsråd (Application to the Publishing Committee of the Natural Science Research Council (NFR) for a publishing grant), 21 August 1952.

22 LUB, AMS, vol. 21, Robert Larsson to John A. Nannfeldt, 4 September 1946; LUB, AMS, vol. 39, Application to NFR for a publishing grant, 30 September 1959. Of 725 subscribers in 1959, 612 were from countries outside Scandinavia.

23 LUB, AMS, vol. 39, Arne Müntzing to Statens naturvetenskapliga forskningsråds publiceringsnämnd (the NFR Publishing Committee), 2 December 1955.

24 LUB, AMS, vol. 6, Robert Larsson to [the Board of the Mendelian Society], 28 December 1919.

25 After Larsson’s retirement, the Mendelian Society arranged a pension for him as part of the NFR Publishing Committee publication grant.

26 Unfortunately, Larsson’s correspondence is not held by the *Hereditas* archive nor any other institutional archive. Part of it is known to be in other persons’ archives..

27 LUB, Albert Levans arkiv (Albert Levan’s Archive), vols. 34–35, Correspondence between Robert Larsson and Albert Levan, 1937–1951.

28 LUB, AMS, vol. 4, MS Board minutes, 9 December 1954.

29 LUB, AMS, vol. 4, MS Board minutes, 20 May 1954, 19 December 1954.

30 LUB, AMS, vol. 4, MS Board minutes, 10 January 1956.

31 When Müntzing became editor, the committee included Johan Rasmusson, Åke Gustafsson, Gert Bonnier, and Erik Essen-Möller.

32 LUB, Arne Müntzings arkiv (Arne Müntzing’s Archive) (AMA), Mogens Westergaard to Arne Müntzing, 29 October 1954.

33 LUB, AMS, vol. 4, MS Board minutes, 15 November 1954; Arkivcentrum Syd, Lund (ACS), Lunds universitetsarkiv (Lund University Archive), Genetiska institutionens arkiv (Archive of the Department of Genetics) (ADG), copy of Arne Müntzing to Mogens Westergaard, 19 November 1954.

34 LUB, AMA, vol. ‘Nordisk genetikerförening’, Love for Nordisk Genetikerforening (Statutes of the Scandinavian Association of Geneticists).

35 LUB, AMS, vol. 37, Arne Lundqvist to Nordiska publiceringsnämnden för naturvetenskap (the Scandinavian Publishing Board in Science) (NOP-N), 24 April 1984.

36 LUB, AMS, vol. 4, MS Board minutes, 19 October 1970.

37 LUB, AMS, vol. 4, MS Board minutes, 29 July 1971.

38 LUB, AMS, vol. 21, PM till tablå över tidskriften *Hereditas* verksamhet under 3-årsperioden 1978–1980 (Appendix to the application to NOP-N for a publishing grant), 10 September 1979. No grant was applied for, the application was drafted solely to inform NOP-N.

39 LUB, AMS, vol. 21, Åke Gustafsson and Arne Lundquist to Rektorsämbetet, Lunds universitet (the Vice-Chancellor of Lund University), 27 July 1977.

40 Ibid.

41 LUB, AMS, vol. 39, Mats Linder, ‘En svensk utredning om vitenskapelige forskningsresultater, deres tilrettelegging, spredning og lagring’ (A Swedish report on scientific research results, their facilitation, dissemination and storage), *NOP-Nytt,* 5 (1976) 5–6.

42 LUB, AMS, vol. 39, Arne Müntzing, Anders Martinsson, Per Brinck, and Stig Falk to Publiceringsnämnden, Statens Naturvetenskapliga forskningsråd (the NFR Publishing Committee), 14 January 1977.

43 LUB, AMS, vol. 21, Appendix to the application to NOP-N for a publishing grant, 10 September 1979.

44 LUB, AMS, vol. 22, PM till tablå över tidskriften *Hereditas* verksamhet under 3-årsperioden 1989–1991 samt till ansökan om publiceringsanslag för årgång 1991 (Appendix to the application to NOP-N for a publishing grant), 12 September 1990.

45 Ibid.

46 LUB, AMS, vol. 36, Skiss till *Hereditas* självbeskrivning (Draft of *Hereditas* mission statement) [1987].

47 LUB, AMS, vol. 36, Cover of *Hereditas* issue 110/1.

48 LUB, AMS, vol. 22, Yearly applications to NOP-N for publishing grants, 1991–1995.

49 LUB, AMS, vol. 36, Utredning om *Hereditas* genomförd våren 1994 (Report on *Hereditas*, spring 1994) by Roland von Bothmer.

50 Mendelian Society of Lund’s current archive (MS Lund Documents), Minutes, 16 September 1999.

51 MS Lund Documents, Minutes, 17 October 2000.

52 MS Lund Documents, Board minutes, 10 January 2001.

53 MS Lund Documents, Board minutes, 28 February 2001, 18 June 2001.

54 MS Lund Documents, Board minutes, 22 August 2002, 26 February 2003, 12 June 2003.

55 MS Lund Documents, Minutes, 3 March 2004.

56 MS Lund Documents, Minutes, 26 May 2004.

57 Personal communication with Andrea Pillmann, 16 October 2020.
