# Correction to: A century of *Hereditas*: from local publication to international journal

**DOI:** 10.1186/s41065-021-00173-1

**Published:** 2021-02-02

**Authors:** Anna Tunlid, Ulf Kristoffersson, Fredrik Åström

**Affiliations:** 1 Department of Arts and Cultural Sciences, Division of History of Ideas and Sciences, LUX, Lund University, Box 192, SE-221 00 Lund, Sweden; 2 Department of Laboratory Medicine, Division of Occupational and Environmental Medicine, Lund University, Scheelevägen 2, SE-223 63 Lund, Sweden; 3 Department of Laboratory Medicine, Division of Clinical Genetics, Lund University, Universitetssjukhuset, SE-221 85 Lund, Sweden; 4 Lund University Library, Lund University, Box 3, SE-221 00 Lund, Sweden

**Correction to: Hereditas 157, 50 (2020)**

**https://doi.org/10.1186/s41065-020-00164-8**

Following publication of the original article [1], an error was identified in the Funding section.

The updated Funding is given below and the changes have been highlighted in bold typeface.

The study was supported by Erik Philip-Sörensens stiftelse för främjande av genetisk och humanistisk vetenskaplig forskning (Erik Philip-Sörensen Foundation for the promotion of genetic research and humanistic studies) and **Svenska Sällskapet för Medicinsk Genetik (Swedish Association for Medical Genetics)**. Open Access funding provided by Lund University.
